# Supplementary material for: Assessing Protein Content and Dimer Formation in the Bevacizumab Reference Product and Biosimilar Versions Marketed in Spain
Source: Pharmaceutics. 2024 Nov 26;16(12):1520. doi: 10.3390/pharmaceutics16121520 (PMC11728500; doi:10.3390/pharmaceutics16121520)

Chromatogram #1: Originator product (Avastin®). Concentration = 50µg/mL

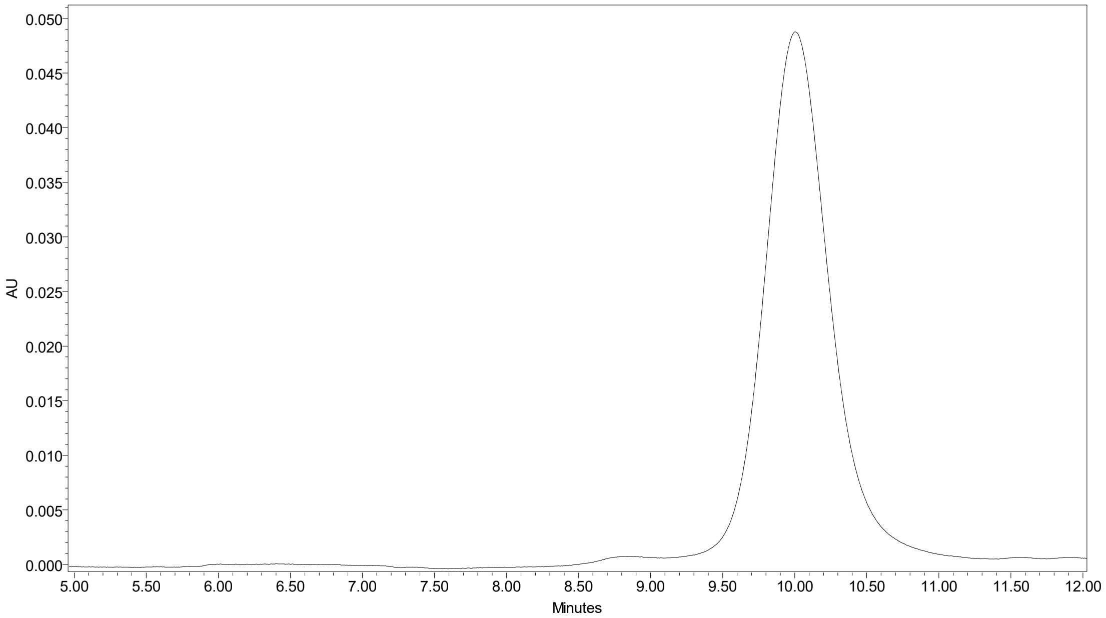

Chromatogram #2. Oyavas®. Concentration = 50µg/mL.

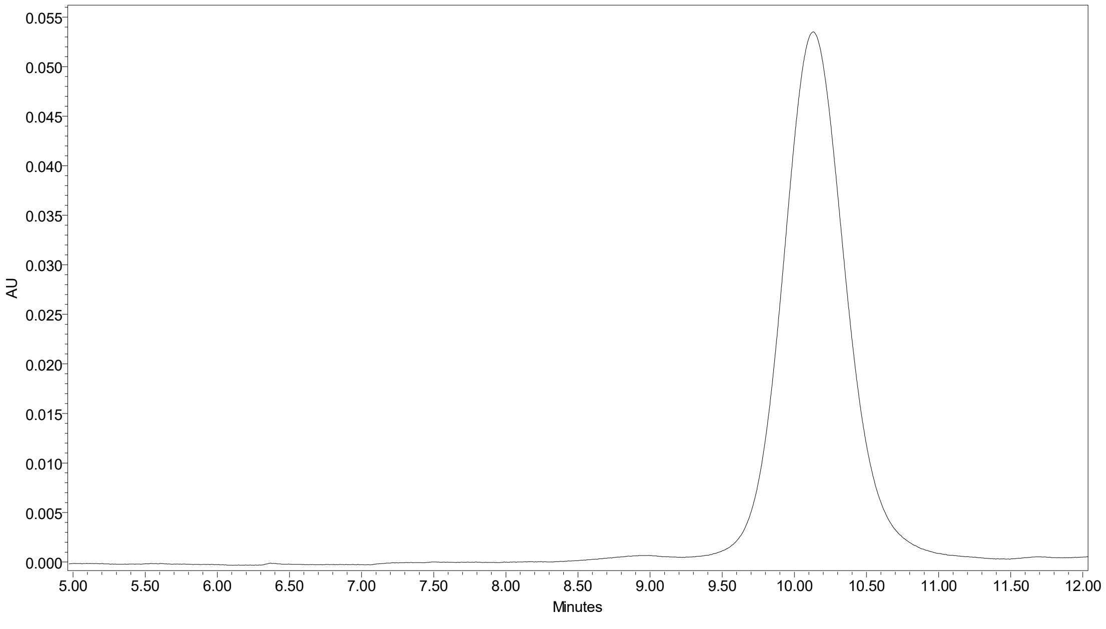

Chromatogram #3: Alymsys®. Concentration = 50µg/mL.

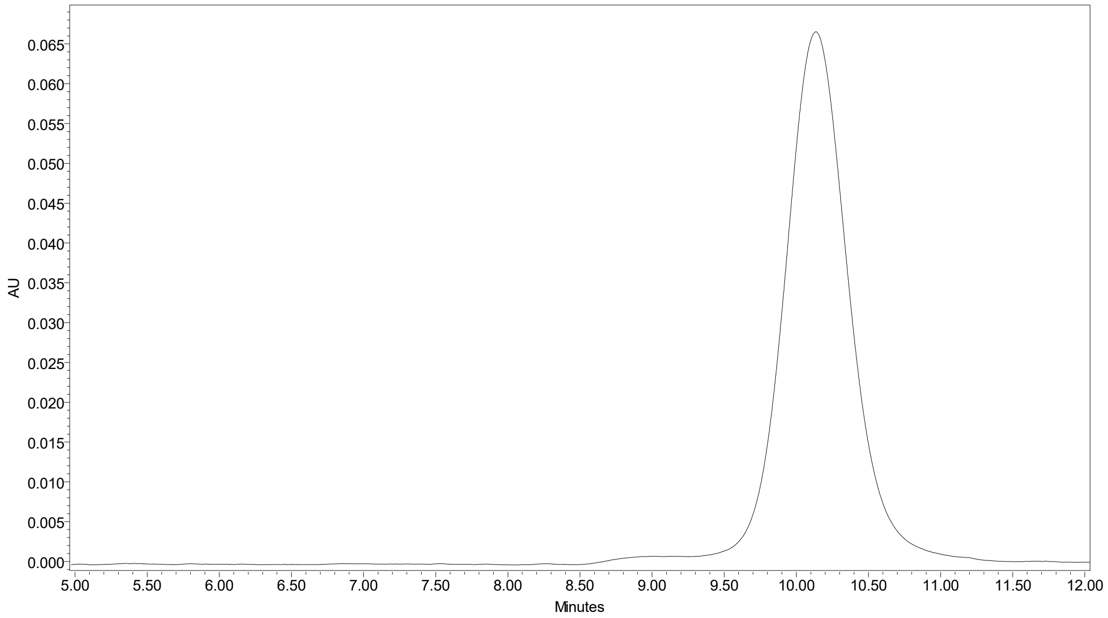

Chromatogram #4: Vegzelma®. Concentration = 50µg/mL.

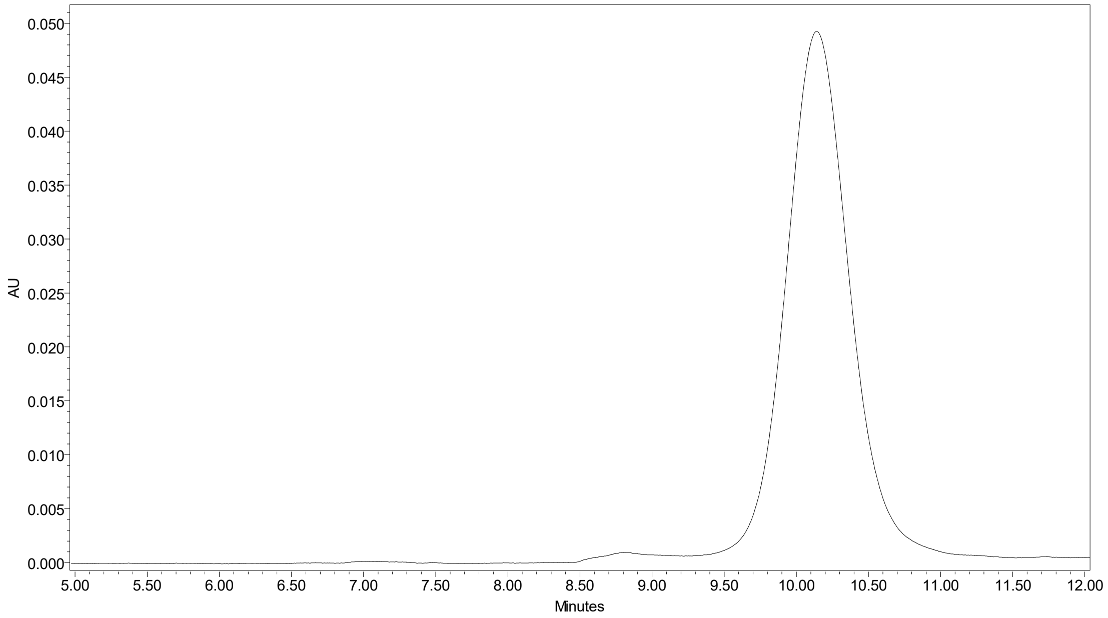

Chromatogram #5: Vegzelma®. Concentration = 25µg/mL.

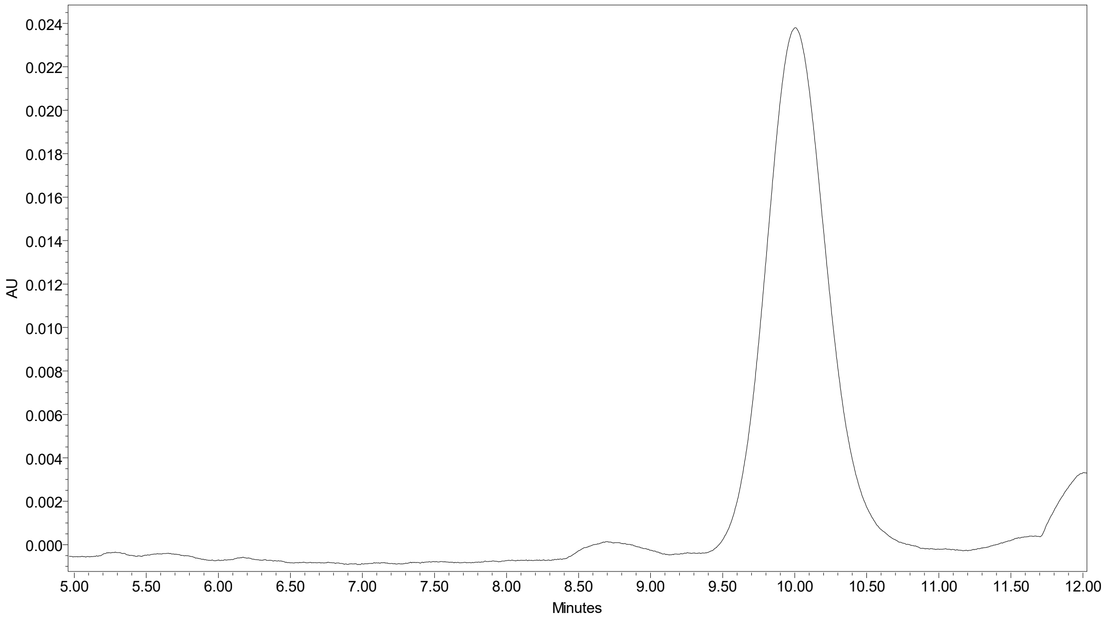

Supplement: Supplementary file 1 [file pharmaceutics-16-01520-s001.zip › SupplementaryMaterial_Cromatograms.pdf]
